# Revascularization of chronic total occlusion coronary artery and cardiac regeneration

**DOI:** 10.3389/fcvm.2022.940808

**Published:** 2022-08-25

**Authors:** Ruoxi Liao, Zhihong Li, Qiancheng Wang, Hairuo Lin, Huijun Sun

**Affiliations:** ^1^Department of Clinical Medicine, Dalian Medical University, Dalian, China; ^2^State Key Laboratory of Organ Failure Research, Department of Cardiology, Nanfang Hospital, Southern Medical University, Guangzhou, China; ^3^Department of Clinical Pharmacology, College of Pharmacy, Dalian Medical University, Dalian, China

**Keywords:** coronary chronic total occlusion, percutaneous coronary intervention, cardiac regeneration, cardiac remodeling, angiogenesis, optimal medical therapy

## Abstract

Coronary chronic total occlusion (CTO) contributes to the progression of heart failure in patients with ischemic cardiomyopathy. Randomized controlled trials demonstrated that percutaneous coronary intervention (PCI) for CTO significantly improves angina symptoms and quality of life but fails to reduce clinical events compared with optimal medical therapy. Even so, intervening physicians strongly support CTO-PCI. Cardiac regeneration therapy after CTO-PCI should be a promising approach to improving the prognosis of ischemic cardiomyopathy. However, the relationship between CTO revascularization and cardiac regeneration has rarely been studied, and experimental studies on cardiac regeneration usually employ rodent models with permanent ligation of the coronary artery rather than reopening of the occlusive artery. Limited early-stage clinical trials demonstrated that cell therapy for cardiac regeneration in ischemic cardiomyopathy reduces scar size, reverses cardiac remodeling, and promotes angiogenesis. This review focuses on the *status quo* of CTO-PCI in ischemic cardiomyopathy and the clinical prospect of cardiac regeneration in this setting.

## Introduction

Due to the limited proliferation potential of cardiomyocytes, injured mammalian hearts do not regenerate adequately but instead develop fibrosis and scarring, leading to heart failure, arrhythmia, and even death. Ischemic cardiomyopathy (ICM) with coronary artery chronic total occlusion (CTO) accelerates the progression of heart failure, which is the leading cause of death worldwide. Despite the development of optimal medical therapy (OMT) and interventional and surgical strategies, the morbidity and mortality of patients with ICM remain relatively high. CTO is associated with a negative impact on long-term prognosis (1), and CTO lesions in a non-infarct-related artery are a high-risk factor for mortality after acute myocardial infarction (AMI) (2). Under such circumstances, it is reasonable to consider that revascularization of the occluded coronary artery would improve the prognosis of patients with CTO. However, several randomized controlled trials (RCT) demonstrated that percutaneous coronary intervention (PCI) for CTO significantly improves angina symptoms and quality of life but fails to reduce clinical events such as mortality, myocardial infarction (MI), stroke, and repeat revascularization rates compared with OMT (3–7). Nevertheless, support for CTO-PCI remains high in clinical practice worldwide. In addition to improving quality of life, we speculate that revascularization should be a premise for further regenerative therapy to improve the prognosis of ICM.

In ICM with CTO, the presence of myocardial hibernation is a primary reason for considering revascularization therapy (8). It is believed that restoring blood flow in the infarcted or ischemic area is important for the repair of myocardial injury. Otherwise, cardiomyocytes are lost quickly or gradually, causing myocardial fibrosis and arrhythmia and leading to heart failure. Accordingly, there has been great support for CTO-PCI or coronary artery bypass grafts (CABGs) over the past two decades. Because fibrotic scar formation often occurs in patients with CTO, restoration of blood flow alone is not able to replace fibrotic scars with cardiomyocytes. In addition to heart transplantation, we believe that effective regenerative therapy combined with the opening of the CTO and OMT would be an optimal approach for curing ICM with CTO.

Cardiac regeneration is a research hotspot that has developed rapidly, with an annual increase of more than 1000 publications in recent years (9). Although substantial progress has been made in experimental studies and various strategies have been developed to induce cardiac regeneration, these interventions still lack adequate success for use in the clinic. In addition to the low efficiency of current regenerative therapy, one contributing factor may be that many efforts have focused primarily on generating cardiomyocytes, with less attention to simultaneous angiogenesis. To maintain the survival and growth of regenerated cardiomyocytes, blood supply to the cells is necessary for oxygen transfer, nutrient absorption and removal of metabolic waste.

Angiogenesis in the heart is formed from preexisting coronary vessels (10). Effective vascular regeneration is critical for enabling the survival of transplanted or regenerated cells. The absence of clinically applicable means of (re)generating vessels is one of the main obstacles in cell replacement therapy (11). The vasculature could also provide important cues for stem cell-derived tissues, which remain immature *in vitro* and require an *in vivo* environment for maturation. Therefore, the role of an appropriate vasculature goes beyond integration with the host system and blood perfusion, and implementing effective vascularization strategies is critical for the success of regenerative medicine. The effects of most cell therapies are mediated by paracrine signaling rather than replacement of lost cardiomyocytes, mainly through the induction of angiogenesis and immunomodulation (9). Thus far, cell-based therapies have delivered unsatisfactory results, prompting the search for cell-free alternatives that can induce the heart to repair itself through cardiomyocyte proliferation and angiogenesis. It seems reasonable to open the occluded arteries as preexisting vessels for angiogenesis and nutrient delivery to the regenerated cardiomyocytes.

## Poor prognosis of coronary chronic total occlusion

Coronary CTO, which is defined as a complete luminal obstruction of a native coronary artery for ≥ 3 months, has been diagnosed in nearly 20% of patients with coronary artery disease (12, 13). In contrast to patients with non-occlusive coronary artery disease, patients with CTO usually have severe comorbidities, such as diabetes mellitus, hypertension, peripheral vascular disease and prior MI (14). CTO can be considered the final stage of obstructive coronary artery disease and is associated with a negative impact on long-term prognosis (1). An undiagnosed or untreated acute thrombotic event is regularly the origin of CTO development, which is supported by electrocardiographic evidence of pathological Q-waves corresponding to the myocardial territory subtended by an occluded artery in one-quarter of patients (12). However, the majority of patients with a CTO have not experienced previous MI (12). In those patients, the occlusion seems to be the result of long-term gradual luminal narrowing allowing for recruitment of collaterals to the occluded vessel. The recruitment of collaterals has a protective role by supplying myocardial blood flow to the CTO territory and thereby preventing acute myocardial ischemia (15).

The myocardial territory supplied by a CTO is a proarrhythmogenic milieu due to the heterogeneity in repolarization and is characterized by scar tissue, hibernating myocardium, and residual ischemia even in the presence of collateral circulation (16). The presence of concurrent CTO is a strong predictor for both short-term and long-term mortality. Patients with a CTO and an implantable cardioverter defibrillator for prevention of sudden cardiac death have a higher incidence of shocks than patients with ICM without a CTO (17). CTOs in a non-infarct-related artery (non-IRA) are present in 10% of patients with ST elevation MI (STEMI) and 23.5% of patients with MI and multivessel disease complicated by cardiac shock (12, 18). The presence of a concomitant CTO in those patients with STEMI is responsible for a higher 30-day event rate and poor long-term prognosis (19). The prognosis especially deteriorates when the occluded vessel receives collateral flow from the IRA (20). In the HORIZONS-AMI trial reported by Claessen et al., patients with a non-IRA CTO were significantly less likely to achieve satisfactory postprocedural reperfusion flow and less frequently achieved complete ST-segment resolution than patients without a CTO (21). Analyses from three clinical trials (HORIZONS-AMI, CULPRIT-SHOCK and TAPAS) demonstrated that multivessel disease with CTO in a non-IRA increases the risk of death for 1 month to 3 years by approximately twofold (HR: 1.63–2.88) (18, 21, 22). CTO has also been reported to worsen the prognosis of patients with type 2 diabetes. Compared to patients without CTO, patients with diabetes and CTO had higher myocardial jeopardy scores and higher 5-year mortality rates than non-CTO patients (23).

The poor prognosis of a concurrent CTO in ICM patients suggests that revascularization therapy of occluded arteries should be highly effective, which is one of the reasons that intervening physicians actively perform CTO-PCI.

## Limited clinical benefits of percutaneous coronary intervention for chronic total occlusion

Ischemic cardiomyopathy is one of the most common causes of congestive heart failure. Accumulating evidence indicates that hibernating myocardium is present in the blood supply region of an occluded artery. Evaluation of viable myocardium can be fundamental for planning myocardial revascularization. Even if excellent collateral circulation develops, symptomatic patients with a CTO usually have a persistent ischemic zone, evidenced by lower fractional flow reserve of the myocardium supplied by a CTO (24). Cardiac magnetic resonance (CMR) can be used to identify inducible myocardial ischemia and viability in the perfusion territory of the artery with CTO; thus, it is believed that CMR is helpful for selecting patients likely to benefit from revascularization (25). In a prospective study of 50 consecutive CTO patients undergoing CMR, Bucciarelli-Ducci et al. reported that CTO recanalization reduces ischemic burden, favors reverse remodeling, and improves quality of life for patients, showing CMR evidence of significant myocardial inducible perfusion defects and viability (26). Similar findings were also found in STEMI patients with CTO (EXPLORE trial) (27).

**FIGURE 1:**
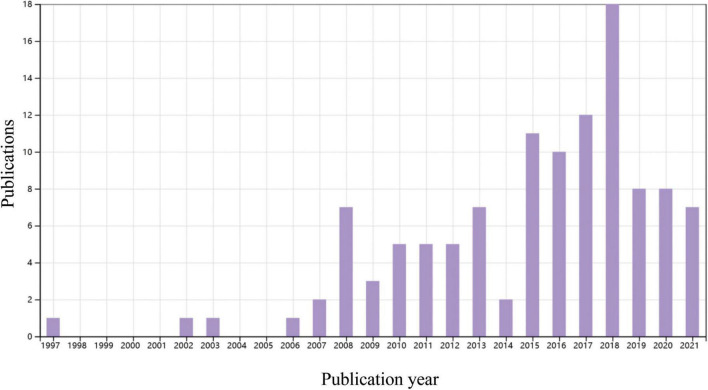
A time course of publications concerning clinical trials of percutaneous coronary intervention (PCI) or coronary artery bypass graft in patients with coronary chronic total occlusion (CTO). Data source: Web of Science, searched with the topics (CTO or “chronic total occlusion”) AND [(PCI or “percutaneous coronary intervention”) OR CABG or “coronary artery bypass graft”] and then refined by document types “Clinical Trial” and “Articles.”

Percutaneous coronary intervention for CTO has been extensively performed worldwide in the last 2 decades. In the Web of Science database, approximately 4000 papers on CTO-PCI could be found up to April 2022. Of them, only 114 articles were related to clinical trials, and the publication numbers peaked in 2018 (Figure 1). A recent meta-analysis reported by Khan et al. compared the clinical effects of CTO-PCI versus OMT from 2006 to 2019 (3). The authors included a total of 16 studies with 11,314 patients. Observational studies showed that CTO-PCI was associated with lower mortality (OR: 0.45) and cardiac deaths (OR: 0.58) than medical therapy alone, but in RCTs, no significant differences in major adverse cardiac events (MACEs) (OR: 0.71, *P* = 0.54), myocardial reinfarction (OR: 0.71, *P* = 0.54), stroke (OR: 0.61, *P* = 0.14), or repeat PCI (OR: 1.28, *P* = 0.16) were observed (3). The possible explanations for the inconsistency of the above results are as follows: First, all the RCTs (Euro CTO, REVASC trials, EXPLORE, DECISION-CTO) included in this analysis were underpowered due to slow enrollment rates and a high crossover rate introducing significant selection bias. Second, majority of these trials involved enrollment of a minimally symptomatic population with relatively lower angina scores, and a better comorbidity index. Although not statistically significant, an average 30–40% risk reduction for MACEs, reinfarction and stroke by CTO-PCI was very impressive. It is reasonable to expect that CTO-PCI would be superior to OMT alone if a sufficiently large sample size and adjunctive regenerative therapy were guaranteed. To date, only 4 RCT comparing clinical prognosis between CTO-PCI and optimal or routine medical therapy alone have been published (Table 1). The DECISION-CTO, EXPLORE, EUROCTO, and IMPACTOR-CTO trials included 417, 150, 259, and 39 PCI patients with procedure failure rates of 9.6, 27, 13.4, and 17%, respectively (4–7). The comparisons among PCI and OMT studies for CTO in RCTs and observational studies were list in Table 2.

**TABLE 1:** Major Findings of the Published RCTs comparing PCI vs. OMT in CTO patients.

Study	Decision-CTO (4)	Explore (5)	Euro-CTO (6)	Impactor-CTO (7)
		Europe and Canada	Europe	
Location and design	Asia	Multicentre RCT (14	Multicentre RCT (28	Russia
	Multicentre RCT (19 centres)	centres)	centres)	Single-centre RCT
N patients	834	304	396	72
Enrolment period	From March 2010 to September 2016	From November 2007 to Apr-15	From March 2012 to May-15	From October 2010 to Apr-14
PCI: OMT	1:1 (*n* = 417:398)	1:1 (*n* = 150:154)	2:1 (*n* = 259:137)	1:1 (*n* = 39:33)
Study population	Patients with a de novo CTO located in a proximal to mid-epicardial coronary artery with a reference vessel diameter of >2.5 mm	Patients with STEMI treated with PCI with a non-infarct-related CTO	SCAD CTO patients with symptoms and/or ischaemia and viability	Patients with isolated dominant RCA CTO and stable angina
Follow-up period	3 years	4 months	1 year	1 year
Primary endpoint	Death, MI, stroke, or any revascularization	LVEF and LVEDV by CMR	QoL (SAQ, EQ-5D)	AMIB by adenosine stress CMR
Primary end point to window follow-up rate	815/834(*n* = 97.7%)	302/304(*n* = 99.3%)	396/396(*n* = 100%)	72/72(*n* = 100%)
Mean J-CTO score	2.1 ± 1.2	2 ± 1	1.82 ± 1.07	1.92 ± 0.86
CTO Success rate	90.60%	73.00%	86.60%	83.00%
Positive/negative RCT	Positive	Negative	Positive	Positive
Major findings	PCI OMT	PCI OMT	PCI OMT	PCI OMT
MACE	No difference	No difference	No difference	No difference
	HR:1.03			
QOL	No difference	N/A	Better	Better
Ischaemia reduction	N/A	N/A	N/A	Better
LVEF and LVEDV	N/A	No difference	N/A	N/A

AMIB, decrease in myocardial ischaemia burden; CMR, cardiac magnetic resonance; CTO, chronic total occlusion; EQ-5D, EuroQol 5 dimensions questionnaire; J-CTO, Japanese chronic total occlusion; LAD, left anterior descending; LVEDV, left ventricular end-diastolic volume; LVEF, left ventricular ejection fraction; MACCE, major adverse cardiac and cerebrovascular events; MACE, major adverse cardiovascular events; MI, myocardial infarction; OMT, optimal medical therapy; PCI, percutaneous coronary intervention; QoL, quality of life; RCA, right coronary artery; RCT, randomized controlled trial; SAQ, Seattle Angina Questionnaire; STEMI, ST-segment elevation myocardial infarction.

**TABLE 2:** Studies of PCI vs. OMT for chronic total occlusion.

Study	Design	Study population	Patients (N)		Study period	Follow-up period	Primary endpoint	Major findings
			PCI	OMT				
Henriques et al. (5)	Multicenter RCT	Patients with STEMI Treated with PCI with a non-infarct-related CTO	148	154	2007–2015	4 months	LVEF and LVEDV by CMR ΔMIB by	No significant difference in MACE between both arms
Obedinskiy et al. (7)	Single-center RCT	Patients with isolated Dominant RCA CTO and stable angina	39	33	2010-2014	1 year	adenosine Stress CMR	ΔMIB was significantly higher in the PCI group in comparison with the OMT group; No QoL parameters improved in the OMT group; No significant difference in MACE-free survival between the PCI and OMT groups
Werner et al. (6)	Multicenter RCT	SCAD CTO patients						Greater improvement of SAQ subscales was observed
		Symptoms and/or ischaemia and viability	259	137	2012-2015	1 year	QoL (SAQ, EQ-5D)	with PCI as compared with OMT for angina frequency and quality of life.
Lee et al. (4)	Multicenter RCT	Patients with a de novo CTO located in a proximal to mid-epicardial coronary artery with a reference vessel diameter of>2.5 mm	417	398	2010-2016	3 years	Death, MI, stroke, or any revascularization	The primary endpoint MACE at 3 years in the intention-to-treat population of patients with a CTO was 20.6% in PCI group as compared to 19.6% in the optimal medical therapy group.
Arslan et al. (89)	Single-center Ret, Ob	Patients determined to have a CTO in at least one coronary artery Patients treated by	117	115	1999-2003	32±12 months	All-cause death	No difference of rates of STEMI and stroke in between two groups
Valenti et al. (90)	Single-center Ret, Ob	successful primary PCI TIMI grade 3 flow andresidual infarct artery stenosis <30%)	58	111	2003-2012	3 years	1- year and 3-year cardiac survival.	The 1-year cardiac mortality rate was 1.7% in the successful CTO-PCI group and 12% in non attempted or failed C 1 O-PCI; Successful C 1 O-PCI was an independent predictor of 3-year cardiac survival.
Lawdinec et al. (91)	Single-center Pro, Ob	Patients with an occluded coronary artery	405	667	2002-2007	5 years	All-cause mortality, MI, MACE	All-cause mortality at 5 years was 11.6% for CTO PCI and 16.7% for medical therapy; The composite of 5-year death or myocardial infarction occurred in 13.9% of the CTO PCI group and 19.6% in the medical therapy group
Jang et al. (92)	Single-center Ret, Ob	Patients with at least 1 CTO detected on diagnostic coronary angiography and symptomatic angina Patients showing at coronary angiography	502	236	2003-2012	42 months	MACE. Cardiac death, repeat revascularization, MI	Lower incidence of cardiac death and MACE in the revascularization group compared with themedication group
		>1 CTO in a main coronary artery (vessel size >2.5 mm)	776	826	2008-2009	1 year		Patients undergoing PCI showed lower rate of Major adverse cardiac and cerebrovascular events and cardiac death in comparis on with those treated with medical therapy
Tomasello et al. (93)	Multicenter Pro, Ob						MACE, Stroke, Cardiac death, MI	
Hwang et al. (94)	Single-center Ret, Ob	Patients with at least 1 CTO and symptomatic angina	288	147	2003-2012	47.6 months	death, repeat revascularization, MI	No significant difference between the OMT group and PCI group with respect to MACE frequency or cardiac death.
Yang et al. (95)	Single-center Ret, Ob	Patients with at least 1 CTO and symptomatic angina	883	664	2003-2012	45.8 months	Cardiac death, All-cause mortality, MI, MACE	No significant difference in the rate of cardiac death between the OMT and PCI groups.
Shuvy et al. (96)	Multicenter Ret, Ob	patients with obstructive CAD defined as stenosis >70% in severity in any major epicardial coronary vessel or >50% in the left main artery	266	849	2012-2013	745 days	Composite of mortality and hospitalization for MI	The rates of mortality or MI in patients with CTO who were treated medically was 11.7%, which were significantly higher than in patients who were treated by CABG or by PCI.
Choi et al. (97)	Single-center Pro, Ob	patients who had at least 1 CTO lesion in the epicardial vessel and 2 or 3 Rentrop collateral grade flow	305	335	2004-2015	5 years	All-cause mortality and hospitalization	CTO-PCI group had a lower hazard of myocardial infarction and the composite of total death or myocardial infarction.
Guo et al. (98)	Single-center Ret, Ob	Patients with at least 1 CTO and symptomatic angina	125	201	2008-2010	47.2±20 months	for MI, MACE, TVR, TLR, change in LVEF MACE, Cardiac death	No significant difference between the 2 groups with respect to the prevalence of MACE.
Choo et al. (99)	Multicenter Pro, Ob	Patients with at least 1 CTO	424	474	2004–2010	2.2 years	All- cause mortality, MACE, coronary revascularization either PCI or CABG, Recurrent MI	The primary end point of all-cause mortality was significantly reduced in CTO-PCI group as compared to medical group.
Rha et al. (100)	Single-center Pro, Ob	Patients were diagnosed with significant coronary artery disease	412	410	2004–2015	5 years	death, MI and MACE: composite of total revascularization either PCI or CABG.	Successful CTO PCI with DESs was associated with a higher risk of repeat PCI for the target vessel but lower incidence of death or MI.
Choi et al. (101)	Single-center Ret, Ob	Patients with CTO of a coronary artery	388	343	2004–2015	5 years	MACE, total death, MI, TVR, T LR and NTVR.	The 5-year cumulative incidence of MACE was similar between the treatment groups regardless of target vessel. The 5-year cumulative incidence of the composite of total death or myocardial infarction was significantly lower after PCI than after OMT or failed PCI in the LCx and RCA groups, but not in the LAD group.
Juricic et al. (102)	Single-center Pro, Ob	Patients with CTO of one coronary artery	50	50	2015–2017	275 ± 88 days	QoL (SAQ)	Patients in the PCI group reported less physical activity limitations, less frequent angina episodes, better QoL, greater treatment satisfaction, and borderline differences in angina stability compared to patients in he OMT group.

AMIB, decrease in myocardial ischaemia burden; CMR, cardiac magnetic resonance; CTO, chronic total occlusion; CTO-PCI, chronic total occlusion-per- cutaneous coronary intervention; DES, Drug Eluting Stent; EQ-5D, EuroQol 5 dimensions questionnaire; LAD, left anterior descending artery; LCx, left circumflex artery; LVED, left ventricle end diastolic volume; LVEF, left ventricle ejection fraction; MACE, major adverse cardiac events; MI, myocardial infarction; Ob, Observational; OMT, optimal medical therapy; PCI, percutaneous coronary intervention; Pro, prospective; QoL: quality of life; RCA, right coronary artery; RCT, randomized controlled trial; Ret, retrospective; SAQ, Seattle angina questionnaire; STEMI, ST elevation myocardial infarction; TIMI, Thrombolysis in myocardial infarction; TLR, target lesion revascularization; TVR, target vessel revascularization.

In patients with diabetes and concurrent CTO, Khan et al. analyzed the results of early revascularization in 1196 cases and OMT in 1252 cases and demonstrated that OMT was associated with higher all-cause mortality [HR: 1.70, *P* = 0.11] and cardiac mortality (HR: 1.68, *P* = 0.07) and a higher risk of repeat revascularization (HR: 1.62, *P* < 0.00001). Subgroup analysis of OMT vs. PCI demonstrated higher all-cause (HR: 1.98, *P* = 0.0003) and cardiac mortality (HR: 1.87, *P* = 0.06) in the OMT group (28). Similarly, Damluji et al. compared the clinical outcome between 482 diabetic patients with prompt revascularization and 490 patients with intensive medical therapy alone. They found that CTO of coronary arteries is associated with increased mortality in patients treated medically but not in patients treated with revascularization (23).

It is generally believed that CTO-PCI can improve the quality of life of patients even if there is no significant reduction in MACEs. The effects of adjunctive regenerative therapy, as well as those of OMT, in patients who undergo CTO-PCI merit further study.

## Effect of opening CTO on cardiac regeneration

Cardiac regenerative medicine focusing on preclinical studies and early-stage clinical trials is rapidly evolving with novel approaches involving cell-based, cell-free and tissue engineering therapies (29, 30). Several thousand review papers have been published on cardiac regeneration, but few have paid attention to cardiac regeneration in the setting of CTO-PCI.

The main conclusions of the clinical trials of cell-based therapy over the last 2 decades are that the outcomes of cell therapy were neutral or marginally positive regarding clinically relevant end points (31). By reviewing the clinical studies on ICM, Nair et al. concluded that a combined approach of simultaneous revascularization and stem cell therapy appears to produce the maximum benefit in ICM (32). In addition to cell therapy, the activation of cardiomyocyte proliferation *in situ* is a promising approach for replacing lost cardiomyocytes. Although potential interest is switching from an exogenous to an endogenous strategy in basic research, there is no clinical trial on endogenous regenerative therapy for the time being.

In the research field of cardiac regenerative therapy, it is common for clinical trials to recruit patients with patent coronary arteries, while experimental studies utilize animal models without coronary revascularization. There are three routes of cell or regeneration-promoting agent delivery: intracoronary, intravenous and intramyocardial (transendocardial) injection. In the setting of CTO without collateral supply, neither intracoronary nor intravenous routes can work for regenerative therapy before the occluded artery is vascularized. Intramyocardial injection is the preferred delivery route for cell therapy in most clinical trials on ICM (33), while intravenously delivered mesenchymal stem cells could improve left ventricular dysfunction through systemic anti-inflammatory effects in ICM (30, 34). Choudhry et al. used a combination of growth factors and bone marrow cells to treat heart failure in ICM patients who had no further treatment options after receiving OMT and undergoing revascularization. They noted that intramyocardial delivery was more effective in improving left ventricular ejection fraction (LVEF) at 1 year than the intracoronary approach (35). However, the outcome of the intracoronary approach for cell therapy in the majority of clinical trials on ICM was positive (32).

In some early clinical studies without CTO-PCI, intracoronary infusion of stem cells from the patent coronary artery to the distribution areas of the occluded artery by collateral flow was performed. Even in that case, a lower incidence of angina symptoms or an increase in LVEF by stem cell therapy was observed (36, 37). As early as 2005, Erbs et al. performed the first RCT to examine whether intracoronary infusion of circulating progenitor cells exerts beneficial effects in patients after recanalization of CTO (38). The authors noted that intracoronary cell therapy after recanalization of CTO results in an improvement in macro- and microvascular function, evidenced by decreases in the infarct size and number of hibernating segments in the target region, an increase in LVEF by 14%, and a reduction in the amount of myocardium with a perfusion-metabolism mismatch in the treatment group (38, 39). Although the sample size was small (26 patients), their results were encouraging.

**FIGURE 2:**
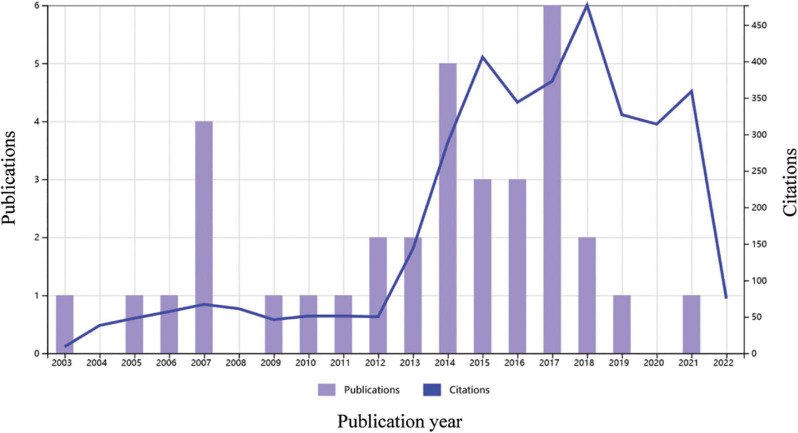
Times cited and number of publications over time related to cardiac regenerative therapy in patients with ischemic heart disease. Data source: Web of Science, searched with the topics “Ischemic cardiomyopathy” AND “Regenerat” and then refined by document types “Articles” and “Clinical Trial,” MeSH headings of “Humans” and “Treatment Outcome,” and excluding document types “Retracted Publications” and “Publication with Expression of Concern.”

Over the last 20 years, 35 articles on clinical trials of regenerative therapy in ICM were published, with peak publication in 2017 and peak citations in 2018 (Figure 2). The average number of citations for each paper was 102, suggesting that regenerative therapy in ICM is a hot topic. Nair et al. summarized 24 completed clinical trials of stem cell therapy in ischemic heart disease, and positive outcomes (improvement in LVEF and reduction in infarct size) were obtained in 13 trials (32), suggesting that regenerative therapy would be a promising approach for resolving heart failure in ICM.

## Simultaneous regeneration of both myocytes and vessels

**FIGURE 3:**
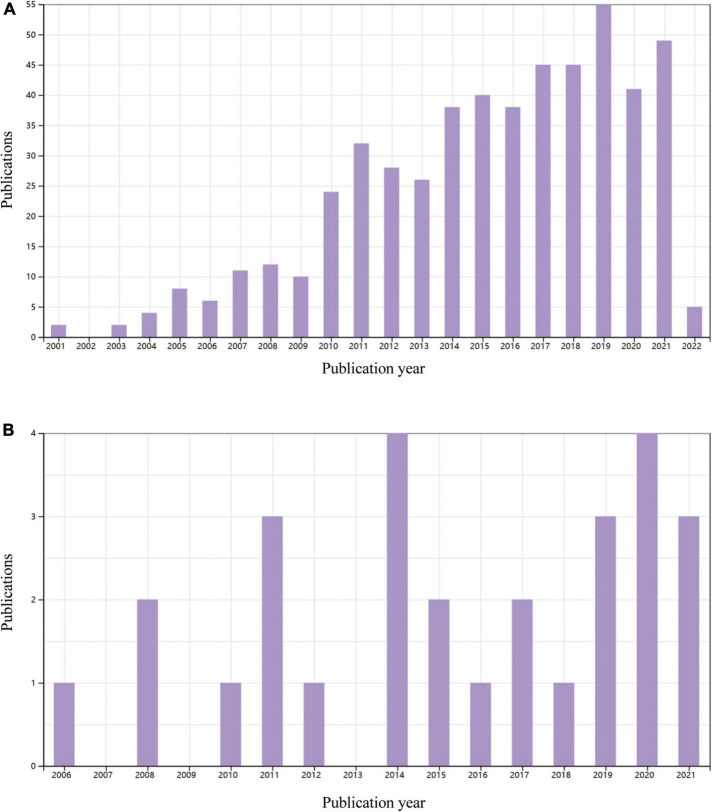
Original publications over time related to cardiac regenerative research in rodents with permanent myocardial infarction of myocardial ischemia/reperfusion in the last 20 years. **(A)** 521 papers on a permanent myocardial infarction model. Data source: Web of Science, searched with the topics “myocardial infarction” AND (”cardiac regeneration” OR “heart regeneration”) and then refined by document type “Articles” and MeSH headings of “Animals.” **(B)** 28 articles using a myocardial ischemia/reperfusion model. Data source: Web of Science, searched with the topic “ischemic/reperfusion” AND (”cardiac regeneration” OR “heart regeneration”) and then refined by document type “Articles” and MeSH headings of “Animals.” Reviews, meeting papers, and editorial materials were excluded from both **(A,B)**.

A water supply is a necessary prerequisite for greening a desert. MI induced by permanent ligation of the left coronary artery in mice usually leads to large ventricular aneurysm (40), which is similar to a desert. It seems incredible to carry out cardiomyocyte regeneration in an aneurysm in the absence of reperfusion. Clinical trials of cardiac regeneration after MI are usually performed in patients with reopening of the infarct-related coronary artery. In contrast, most of the animal studies on cardiac regeneration employed rodent MI models with permanent occlusion of the coronary artery. By searching the Web of Science database, we found more than 2500 original research papers focusing on MI-related heart regeneration in experiments using rodents, while only 28 papers adopted an ischemia/reperfusion model to study cardiac regeneration (Figure 3). Although many encouraging results on cardiomyocyte regeneration have been reported in MI animal models, it is still questionable how the regenerated cells survive without an adequate blood supply. Revascularization or surgical reshaping of the excessively dilated left ventricle would facilitate regenerative therapy (38, 39, 41).

**FIGURE 4:**
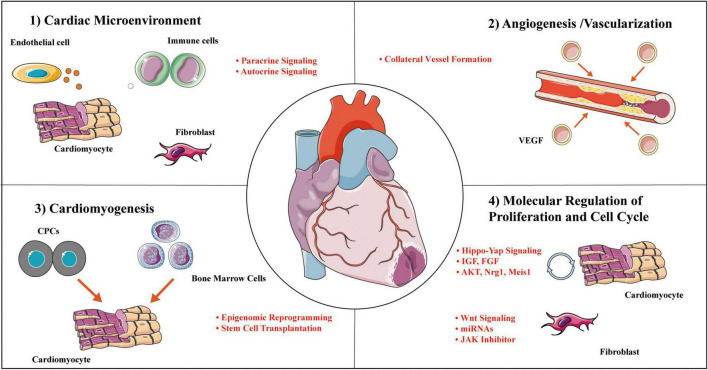
Cardial regeneration involves multiple mechanisms. Representative categories and selected examples of processes to enhance cardial regeneration covered in this review. Mechanisms work independently on a molecular level to collectively mediate concurrent cellular actions of regenerative responses. CPC, cardiac progenitor cell; IGF, insulin-like growth factor; JAK, janus kinase; Meis-1, Meis homobox 1; Mps-1, monopolar spindle 1; VEGF, vascular endothelial growth factor.

Adult mammalian cardiomyocytes have poor proliferative and consequently regenerative potential following injury. The inability to replace lost cardiomyocytes after MI is paralleled by scarring at the injured area. Timely revascularization is an effective treatment to curb cardiac deterioration. Although it is largely unknown the effects and mechanisms of CTO-PCI on cardiac regeneration in patients, the key mechanisms of cardiac repair and regeneration after MI or ischemia/reperfusion clarified in animal studies likely work in patients with CTO-PCI. As summarized in Figure 4, cardiac regeneration may be achieved by way of: (1) alterations in the cardiac microenvironment, (2) angiogenesis/vascularization, (3) stem cell therapy, (4) proliferation and cell cycle molecular regulation. The adult heart consists of cardiac myocytes, endothelial cells (majority representing vascular endothelial cells), fibroblasts, and immune cells. Under physiological conditions, non-cardiomyocytes act on cardiomyocytes through a paracrine mechanism. In the CTO-PCI heart, due to changes in the cardiac microenvironment caused by the restoration of coronary artery blood flow, various cells in the heart act on cardiomyocytes through various mechanisms, promoting myocardial regeneration or reducing cardiomyocyte death, and ultimately improving cardiac function.

Angiogenesis is essential for the repair and regeneration of cardiac tissue after MI. The formation of new capillaries may be of clinical importance in facilitating regeneration in fibrotic cardiac tissue after MI. Vascular endothelial growth factor (VEGF) is a cornerstone cytokine involved in promoting the formation of new blood vessels, and thus has been a focus in the treatment of heart disease (42, 43). VEGF expression is increased in the epicardium and subepicardium cells of the aortic root, and these molecules are thought to regulate endothelial cell penetration into the aorta (44). In addition, direct intravenous injection of VEGF into endothelial cells induces an angiogenic phenotype similar to that found in coronary vessels (45). The delivery of VEGF-A in combination with various stent combinations has also been successful in stimulating angiogenesis and restoring cardiac function (46, 47). Members of the VEGF family are key regulators of the development of blood vessel and lymphatic vessels. Similar to systemic lymphatics, cardiac lymphatics require Vegfr3-Vegfc signaling to develop in genetic models such as Vegfr3^–/–^ and Vegfc± zebrafish (48, 49). It is known that an adult zebrafish can regenerate its injured heart with an early response of coronary revascularization, while disruption of this process by blocking VEGFc signaling leads to impaired cardiomyocyte repopulation (50). VEGFc is secreted by the epicardium and pro-inflammatory macrophages after MI in mice, which drives lymphangiogenesis and extensive remodeling of the cardiac lymphatic network (51). This endogenous response of cardiac lymphatics attempts to maintain the optimal immune cell load necessary for effective tissue repair (52). Thus, disruption of Vedfr3-Vegfc pathway blocks lymphatic response to freeze injury, which leads to inefficient immune cell clearance and increased scar formation. Hence, coronary revascularization holds great therapeutic potential for myocyte regeneration.

At the same time, stem cell therapy is one of the most commonly used treatments for improving cardiac function in clinical studies of ICM after revascularization (Tables 3, 4). Possible mechanisms for its improved cardiac function include myocardial regeneration, angiogenesis, and paracrine activities of the cells. Even in a permanent MI mouse model without revascularization, angiogenesis is usually accompanied by successful myocyte proliferation in response to intramyocardial injection of exosomes secreted by human diosphere-derived cells or embryonic stem cells. (53, 54). Intramyocardial injection at the border zone of MI is not clinically appealing due to its invasive nature. Vandergriff et al. utilized an ischemia/reperfusion rat model to examine the effect of intravenously infused exosomes on cellular proliferation and angiogenesis (55). They noted that cardiac-homing peptide-derived exosomes significantly improved the outcomes of myocyte proliferation and angiogenesis (55). Similarly, systemic injection of regeneration-associated cells in a rat model of ischemia/reperfusion improved cardiac function and enhanced capillary density (56). These findings suggest that the reopening of the IRA is important for targeting exosomes to the infarcted heart. Numerous preclinical studies have shown that exosomes are protective in ischemic heart disease by alleviating myocardial ischemia–reperfusion injury, promoting angiogenesis, inhibiting fibrosis, and facilitating cardiac regeneration (57), further supporting the importance of simultaneously promoting myocyte proliferation and angiogenesis.

**TABLE 3:** Stem cell therapy in ischemic cardiomyopathy after revascularization.

Study	Design	Patients (N)	Cell type	Route of administration	Follow-up		Major findings
					period	Primary Endpoint	
Strauer et al. (83)	Observational	20	BMC	Intracoronary	3 months	Infarct size at 3 month	Decreased infarct size with improvement in LV contractility
Wollert et al. (85)	RCT	60	BMC	Intracoronary	6 months	Global LVEF	6.7% increase in LVEF in the BMC group at 6 months post MI
Schachinger et al. (103)	RCT	204	BMC	Intracoronary	4 months	Global LVEF	5.0% increase in LVEF in the BMC group at 4 months post MI
Lunde et al. (104)	RCT	100	BMC	Intracoronary	6 months	LVEF	No changes between control and BMC groups
Huikuri et al. (105)	RCT	80	BMC	Intracoronary	6 months	Global LVEF	Increased global LVEF and neutral effects on arrhythmia risk
Zhao et al. (106)	RCT	36	BMC	Intramyocardial	6 months	Cardiac function and perfusion	Improved cardiac function and perfusion at 6 months
Ang et al. (107)	RCT	63	BMC	Intramuscular or Intracoronary	6 months	Contractile function	No improvement in contractile function of scar segments
Hirsch et al. (108)	RCT	200	BMC	Intracoronary	4 months	LVEF	No changes in LVEF or volume, mass or infarct size
Traverse et al. (109)	RCT	87	BMC	Intramyocardial	6 months	Global/regional LV function	No improvement in function at 6 months
Hu et al. (110)	RCT	60	BMC	Intra graft	6 months	LV function	Improved LV function No improvement in LV
Patila et al. (111)	RCT	39	BMC	Cell transplantation	1 year	LV systolic function	Systolic function orviability
Can et al. (112)	RCT	79	HUC-MSC/B MC-MNC	Intramyocardial	1 year	Ventricular Remodeling	Ongoing
Nicolau et al. (113)	RCT	121	BMC	Intracoronary	6 months	Mean LVEF	No change in mean LVEF at 6 months

BMC, Bone Marrow Cells; BMC-MNC, Bone Marrow Mononuclear Cells; HUC-MSC, human umbilical cord mesenchymal stem cells; LV, left ventricular; LVEF, left ventricular ejection fraction; RCT, randomized controlled trial.

**TABLE 4:** Pre-clinical studies of stem cell therapy for cardiac regeneration.

Study	Animal Model	MI Model	Cell type	Administration	Timing of cell therapy after MI	Follow-up (weeks)	Effect
Lim et al. (114)	Pig	LAD, I/R	MSC	IC	3 days	4	Increased LVEF and decreased the area of MI
Moelker et al. (115)	Pig	LCX, I/R	BM-MNC	IC	7 days	4	Reduced MI size
Price et al. (116)	Pig	LAD, I/R	MSC	IV	1 h	13	Improved LVEF
Makela et al. (117)	Pig	LCX, I/R	BM-MNC	Surgical	1 h	3	Improved the ejection fraction
Moelker et al. (118)	Pig	LCX, I/R	USSC	IC	7 days	4	No difference in global and regional LV function Reduced fibrosis and inflammatory infiltrate, improved
Qian et al. (119)	Pig	LAD, I/R	MSC	IC	7 days	6	Cardiac function
Valina et al. (120)	Pig	LAD, I/R	MSC/ADSC	IC	1 h	4	Improved LVEF
Yang et al. (121)	Pig	LAD, I/R	MSC	IC	28 days	4	Improved cardiac function
deSilva et al. (122)	Pig	LAD, I/R	BM-MNC	IC	4 days	6	No improve remodelling, contractile function, perfusion or infarct size
Doyle et al. (123)	Pig	LCX, I/R	EPC	IC	2 days	8	Induced cardiomyocyte hypertrophy and increased infarct territory LV mass
Gyongyosi et al. (124)	Pig	LAD, I/R	MSC	TE	16 days	1.5	Reduced MI size
Halkos et al. (125)	Pig	LAD, I/R	MSC	IV	1 h	12	Enhanced early reperfusion augments vasculogenesis, regional perfusion and improved ventricular function
Hashemi et al. (126)	Pig	LAD, I/R	MSC	TE	3 days	8-12	Reduced MI size
Perin et al. (127)	Dog	LAD, I/R	MSC	TE/IC	7 days	2	Increased vascularity and greater functional improvement
Qi et al. (128)	Pig	LAD, I/R	MSC	IC	5 days	4-8	Improved LVEF
Schuleri et al. (129)	Pig	LAD, I/R	MSC	TE	2 days	8	Reduced apoptosis in the infarct zones and improved regional and global LV function
Johnston et al. (130)	Pig	LAD, I/R	CDC	IC	28 days	8	Reduced MI size
Quevedo et al. (131)	Pig	LAD, I/R	MSC	IC	84 days	12	Improved EF, reduced MI size
Schuleri et al. (132)	Pig	LAD, I/R	MSC	Surgical	111 days	12	Reduced infarct size
Wang et al. (133)	Pig	LAD, I/R	MSC	Transcoronary injection	1 h	4	Improved LVEF and cardiac function
Yang et al. (134)	Pig	LAD, I/R	MSC	Surgical	1 h	6	Reduced MI size and improved cardiac function
Jiang et al. (135)	Pig	LAD, I/R	MSC	IC	1 h	13	Improved cardiac function
Arslan et al. (136)	Mouse	LCA, I/R	ESC-MSC	IC	Immediately	4	Reduced MI size, decreased LV dilation, increased cardiac function, decreased ATP loss
Agarwal et al. (137)	Rat	LAD, I/R	CPC	IM	Immediately	4	Improved cardiac function, decreased fibrosis and improved angiogenesis
Gallet et al. (138)	Pig	LAD, I/R	CDC	IM	4 weeks	4	Decreased scar size, LV collagen content and cardiomyocyte hypertrophy, increased vessel density.
Liu et al. (139)	Rat	LAD, I/R	MSC	IM	immediately	1	Decreased apoptosis and MI size, improved cardiac function
Adamiak et al. (140)	Mouse	LAD, I/R	iPSC	IM	2 days	5	Improved cardiac function, decreased apoptosis and hypertrophy, improved angiogenesis
Vandergrif et al. (55)	Rat	LAD, I/R	CDC	Intravenous Injection	1 days	3	Reduced apoptosis, infarct size and improved left ventricle ejection fraction
Ciullo et al. (141)	Rat	LAD, I/R	CPC	Intravenous Injection	Immediately	4	Reduced infarct size and improved left ventricle ejection fraction
Zhao et al. (142)	Mouse	LAD, I/R	BM-MSC	IM	Immediately	3	Decreased MI size and inflammation

ADSC, adipose tissue-derived stem cells; ATP, adenosine triphosphate; BM-MNC, bone marrow mononuclear cells; CDC, cardiosphere-derived cells; CPC, cardiac progenitor cell; EPC, endothelial progenitor cells; ESC, embryonic stem cells; IC, intracoronary infusion; IM, intramyocardial; I/R, ischaemia/reperfusion; iPSC, induced pluripotent stem cells; LAD, left anterior descending artery; LCX, left circumflex artery; LV, left ventricle; LVEF, left ventricle ejection fraction; MI, myocardial infarction; MNC, peripheral mononuclear cells; MSC, mesenchymal stem cells; TE, trans-endocardial injection; USSC, unrestricted somatic stem cells.

Genetic triggers for cell cycle reactivation to drive mitosis in adult cardiac myocytes have been advanced as potential therapeutic targets for cardiac regeneration. For example, Hipo-YAP signaling is critical for the intracellular regulation of cardiomyocyte proliferation. Hippo deficient mouse embryos developed cardiac hypertrophy, high proliferation of cardiomyocytes, and enhanced classical Wnt (wingless-type mouse mammary tumor virus)/β-catenin signaling (58). Yap-conditional knockout neonatal hearts failed to regenerate after MI at postnatal day 2, displayed extensive fibrotic infarct scar and deleterious loss of healthy myocardium, while constitutive Yap activation in adult heart significantly enhanced cardiac regeneration, improved cardiac function (59). The regeneration activity of Yap is partly related to the stimulation of the IGF/Akt/GSK-3β/β-catenin pathway. Another candidate for regulating cardiomyocyte proliferation is MEIS-1 (Meis homobox 1), a homeodomain transcription factor essential for normal cardiogenesis and embryonic hematopoiesis. Loss of MEIS-1 in the adult heart increases the number of cardiomyocytes that enter the cell cycle and increases cytokinesis (60). Moreover, fibroblasts play an important role in cardiac regeneration through myofibroblast transdifferentiation via the WNT signaling pathway. Using genetic engineering, fibroblasts can be induced to differentiate into cardiomyocytes or cardiac pluripotent stem cells with selected miRNA or JAK (Janus kinase) inhibitors (61, 62).

In addition to the above-mentioned cells in the heart, the cardiac rhythm cells have an irreplaceable role in maintaining the normal operation of the heart. Lerchenmüller et al. reported that exercise can induce cardiac regeneration and pathways related to circadian rhythm in mice (63). Although the evidence is limited on relations between myocardial regeneration and cardiac rhythmic cells after revascularization, it can be expected that circadian rhythm plays a crucial role in cardiac regeneration.

## The feasibility of cardiac regeneration after chronic total occlusion revascularization in animals

It is unclear whether endogenous regenerative therapy would be more effective for the prognosis of patients with CTO revascularization. Basic research on CTO is largely limited due to the difficulty in establishing an experimental animal model of CTO that can accept manipulation of CTO revascularization. Animal experiments on myocardial ischemia are mostly performed in young and healthy animals that lack the risk factors and comorbidities that are characteristic of patients suffering from acute or chronic myocardial ischemia. Although there is no animal model that can fully mimic both CTO and CTO revascularization in humans, attempts toward creating an animal model of coronary recanalization would be helpful for seeking and confirming new therapeutic targets as well as clarifying the underlying mechanisms.

A major limitation of atherosclerotic animal models is that atherosclerotic plaques usually occur in the aorta and proximal great arteries rather than in the coronary arteries. It is workable to generate a CTO model by adding environmental stress to gene-targeted mice (64), but it is difficult to perform CTO revascularization in those animals. In 2019, Marino et al. reported a mouse model with atherosclerosis capable of recapitulating coronary plaque disruption, thrombosis, and MI (65). They demonstrated that exposure of the heart of ApoE knockout mice to high pressure could induce myocardial events due to coronary plaque thrombosis and occlusion in 74% of the mice. This model is strikingly similar to patients with coronary artery disease and hypertension, and some of those animals could experience coronary occlusion similar to human CTO. As early as 2002, Braun et al. reported that mice with double knockout of the high-density lipoprotein receptor SR-BI and ApoE exhibit coronary artery occlusion, spontaneous MI and cardiac dysfunction with similarities to those seen in human coronary artery disease, but all of those mice died at 8 weeks of age (66). In addition, ApoE^–/–:^Ins2^+/Akita^ male mice fed a Western diet (hyperglycemic and hyperlipidemic mice) also have coronary atherosclerosis, MI and a significant reduction in lifespan (67), while chronic intermittent mental stress promotes plaque instability and MI in ApoE(–/–)fibrillin-1 (C1039G±) mice (68).

At present, there is no CTO model in large animals that simulates the developmental process of CTO in humans for the following reasons: (1) CTO in coronary arteries cannot be directly induced by surgical methods; (2) it is difficult for the coronary arteries of large animals to form atherosclerotic changes similar to those in humans, especially calcification; (3) the process of CTO also includes the occurrence of inflammatory reactions, which is not easy to achieve in animal models; and (4) although conventional interventional treatments such as balloon dilation and stent placement can cause damage to the coronary endothelium and the formation of neointima in animals, the probability of complete occlusion of the blood vessel is very small (69, 70). For the above reasons, many of the reported CTO models use the peripheral blood vessels of animals, which are not feasible for the study of cardiac regeneration.

The model animals in the basic research on interventional cardiology include mice, rats, rabbits, dogs, and pigs, among which the pig and rabbit models are the most commonly used because the response to injury of porcine coronary artery or rabbit femoral artery is closer to that of human coronary arteries, and the choice of surgical approach is more convenient (71). For the CTO model, more damage is needed to cause vascular occlusion. Compared with the miniature pig coronary artery model, the rabbit femoral artery model is relatively simple to establish and costs less. It was reported that the degree of injury in the rabbit femoral artery after balloon strain was very similar to that of the human coronary artery, both of which exhibited tearing of the vascular medium membrane and plaque rupture (72), and this model could simulate many features of human coronary CTO, including early thrombosis, an acute inflammatory response, and vascular remodeling (73, 74).

Coronary occlusion in large animals can be achieved by direct ligation or placement of an artery constrictor (75, 76), but few investigators have tried to reopen the vessels. Suzuki et al. from Japan used bone meal and an absorbable gelatin sponge to establish a coronary artery CTO lesion model in miniature pigs that could simulate the calcification process in human CTO and induce pathological processes such as inflammatory cell infiltration and the formation of bridge collaterals. More importantly, this type of coronary CTO could be reopened by interventional therapy (77). This model should be an ideal animal model that largely simulates human CTO, but there are obstacles to performing experimental studies on post-CTO regeneration of cardiomyocytes in the porcine heart due to the difficulty of gene manipulation and the high cost.

More accurate small-animal models that represent human CTO and heart failure are needed to perform early efficacy testing of novel regenerative therapies. A rodent model of CTO and CTO revascularization would be essential for connecting the basic and clinical research on post-CTO regeneration of cardiomyocytes. However, except for the acute ischemia/acute reperfusion model, no rodent model of CTO/reperfusion is available. It seems reasonable to use an absorbable suture to ligate a coronary artery to partially simulate CTO revascularization in mice or rats. Using a 2-week absorbable suture to constrict the mouse aortic arch, Lao et al. demonstrated that this procedure could cause significant myocardial hypertrophy at 2 weeks and that myocardial hypertrophy almost completely regressed to baseline at 4 weeks after surgery. (78). It is imaginable that absorbable suture ligation can induce complete coronary occlusion in the early stage and allow the coronary artery to reopen after the ligating suture has been absorbed in the late stage. One concern that should be noted is that permanent coronary ligation of the left coronary artery in mice would induce large ventricular aneurysm (40); in that situation, what is the value of reopening the ligated coronary artery? Surgical ventricular restoration to reshape the markedly dilated LV and collapse the large aneurysm would facilitate regenerative therapy (41). In fact, surgical ventricular restoration has repeatedly been suggested as a viable alternative in managing heart failure in select patients with a large LV and refractory heart failure, as it is believed that surgically returning the ventricle to its original dimensions is possible and is associated with favorable outcomes (79). It may be feasible to generate a murine model with a smaller infarct size using absorbable suture ligation, which would facilitate regenerative studies after CTO recanalization.

## Clinical prospects

Ischemic cardiomyopathy is a major contributor to refractory heart failure, which has a poor prognosis. OMT and different coronary revascularization strategies are the mainstays in the management of ICM. Although the role of medications and mechanical circulatory support is ever increasing, cardiac transplantation remains the last hope for treating advanced heart failure. Limited by the small number of available and suitable donor hearts, efficient cardiac regeneration would be an ideal replacement for cardiac transplantation in alleviating heart failure.

Randomized controlled trials including the Decision CTO and the Euro CTO studies did not yield positive results, showing that CTO patients were not able to obtain hard end point improvement (reduction in MACEs) from PCI. However, this should not be misinterpreted to mean that CTO recanalization was an invalid measure (4). Based on clinical practice and relevant guidelines (80), the current indications for interventional treatment of CTO lesions include the following: (1) CTO with symptoms of myocardial ischemia, and CTO with poor angina control that is still present after OMT; (2) non-invasive examination confirmation of the presence of massive myocardial ischemia in the area dominated by the diseased vessels; and (3) coronary angiography showing that the occlusion is suitable for interventional therapy. Application of viability testing is helpful in predicting whether revascularization is able to prevent further damage by protecting the residual viable myocardium from subsequent acute coronary events (81). The J-CTO scoring system summarized based on the success rate of surgery can reflect the difficulty of CTO surgery to a large extent and predict the success rate of surgery (82).

As early as 2002–2004, three studies initiated cell-based therapy by intracoronary injections to treat patients with acute MI after percutaneous transluminal coronary angioplasty (83–85). In 2005, Erbs et al. first reported an intracoronary cell-therapy RCT in patients with coronary CTO but without heart failure after PCI (38). Early-stage clinical trials suggest that cardiac regeneration induced by exogenous cell therapy is effective in improving cardiac function in patients with ICM (32), but there are several shortcomings, such as low efficiency, ventricular arrhythmias, and immune rejection (86). Clinical translation of endogenous regenerative therapy would provide new hope for alleviating heart failure. Revascularization and surgical ventricular reshaping may be beneficial for improving the regenerative environment. Recanalization of CTO would enhance the delivery efficiency of endogenous regenerative factors such as extracellular vesicles and autologous mitochondria (87, 88), promote angiogenesis and deliver nutrients to the proliferated cardiomyocytes.

Chronic total occlusion revascularization, surgery to reshape the excessively enlarged left ventricle and the development of high-efficiency regenerative therapy may hold promise in the future for providing permanent solutions for refractory heart failure in patients with ICM.

## Author contributions

HL and HS: concept design, data interpretation, manuscript writing and revising. RL: data collection, analysis, interpretation, and manuscript writing. ZL and QW: data analysis and interpretation. All authors contributed to the article and approved the submitted version.
